# Microbial Inhibition by UV Radiation Combined with Nisin and Shelf-Life Extension of Tangerine Juice during Refrigerated Storage

**DOI:** 10.3390/foods12142725

**Published:** 2023-07-17

**Authors:** Isaya Kijpatanasilp, Khursheed Ahmad Shiekh, Saeid Jafari, Randy W. Worobo, Kitipong Assatarakul

**Affiliations:** 1 Department of Food Technology, Faculty of Science, Chulalongkorn University, Bangkok 10330, Thailand; ykijpat@gmail.com (I.K.); khursheedahmad.s@chula.ac.th (K.A.S.); saeid.j@chula.ac.th (S.J.); 2 Department of Food Science, College of Agriculture and Life Sciences, Cornell University, Ithaca, NY 14853, USA; rww8@cornell.edu

**Keywords:** bacteriocin, cold storage, fruit juice, hurdle concept, microorganisms, quality

## Abstract

This study evaluated the efficiency of UV radiation doses (4.68–149.76 J/cm^2^) and nisin (50–200 ppm) and their combination in comparison with thermal pasteurization on the microbial inhibition kinetics and physicochemical properties of tangerine juice. It was noted that UV-149.76 J/cm^2^ and nisin (NS) at 200 ppm in conjunction exhibited the highest log reduction in spoilage and pathogenic microbes including *Escherichia coli*, *Lactiplantibacillus plantarum*, and *Saccharomyces cerevisiae*, yeast and molds, and total plate count in tangerine juice. Additionally, the first-order kinetic model provides a better fit for spoilage and pathogenic strains compared with the zero-order model (higher coefficient of determination, R^2^), particularly for *E. coli*. UV and NS showed insignificant effects (*p* > 0.05) on pH, TSS, and TA values compared with pasteurization. However, there were notable differences observed in color analysis, total phenolic compound, total flavonoid content, vitamin C, carotenoid content, and antioxidant activity using DPPH and FRAP assays. The optimized UV + NS samples were subjected to refrigerated storage for 21 days. The results revealed that during the entire storage period, the pH values and the TSS values slightly decreased, and the TA values increased in the treated samples. The UV + NS treatment insignificantly impacted the color properties. The total phenolic, total flavonoid, and carotenoid contents, and vitamin C decreased over time for all sample treatments, whereas the antioxidant properties exhibited varying outcomes, compared with an untreated control and pasteurization. Therefore, UV radiation and nisin (UV-149.76 J/cm^2^ + NS-200 ppm) in combination could serve as a viable alternative to traditional heat pasteurization of fruit juice during cold storage.

## 1. Introduction

As people become more health-conscious, there has been a surge in demand for food and beverages that are both healthy and functional. This trend has led to a rise in the popularity and availability of fresh juices and beverages in supermarkets [1]. Tangerine juice is a well-liked beverage that has several health advantages. It contains significant amounts of vitamin C, which supports a healthy immune system and guards against illnesses and infections. Antioxidants, which are included in orange juice, may aid in preventing cell damage and lowering the risk of chronic illnesses like cancer and heart disease [2]. Therefore, consuming tangerine juice often as part of a balanced diet might be an effective way to enhance general health and wellbeing. However, tangerine juice is a perishable product that is vulnerable to oxidation and microbial growth, which can lead to a decrease in its nutritional content and potential health benefits during storage.

Pasteurization is a preservation method that helps to make the liquid safer for consumption by reducing the risk of spoilage. However, this process can also result in a loss of certain heat-sensitive nutrients and enzymes in the liquid as well as some negative impacts on the sensory qualities of the juice. On the other hand, nonthermal technology is a type of processing using techniques other than heat to preserve foods, including fruit juice. Some of the benefits of nonthermal processing of fruit juice include retention of nutrients, improved flavor and sensory characteristics, and extended shelf life [3]. In order to improve the sensory quality of the finished product while still maintaining microbial safety, ultraviolet irradiation has been researched and developed as a substitute for thermal treatment [4]. In our recent study, we demonstrated that UV treatment of longan juice at a dose of 74.88 J/cm^2^ reduced microbial loads, improved quality characteristics, and extended its shelf life during cold storage [3]. Furthermore, when combined with other hurdle technologies, such as the use of chemicals, high-pressure processing, pulsed electric fields, or ozone treatment, the benefits of UV treatment are magnified. Overall, the combination of UV treatment with other hurdle technologies can offer a comprehensive approach to fruit juice processing, resulting in a safer, higher-quality, and longer-lasting product. Nisin is also a naturally occurring antimicrobial peptide produced by *Lactococcus lactis*. It is a peptide known to inhibit bacterial growth, and is widely used in the food industry. It can also be used in combination with UV treatment for fruit juice processing [5]. Verma et al. [6] concluded that bacteriocins such as nisin are more promising preservatives in food products than their chemical counterparts. The previous study reported that UV-C radiation and nisin could significantly reduce microbial contamination on shrimp without significantly affecting quality parameters of the shrimp such as texture or lipid oxidation [7]. However, their effects on tangerine juice have not been elaborated on yet.

This study examined a new processing approach (hurdle concept) for tangerine juice that combines UV irradiation and nisin treatment, and compares it with conventional pasteurization methods. Kinetic modeling was used to analyze the inhibition of spoilage and pathogenic microorganisms by UV treatment, and the effects of UV + nisin treatment on the physical and chemical properties of tangerine juice were also explored. In addition, changes in quality of UV + nisin treated samples were investigated during refrigerated storage. 

## 2. Materials and Methods

### 2.1. Tangerine Juice Preparation

Tangerines were obtained from a retail market in Bangkok, Thailand. The tangerines were peeled and processed using an Electrolux EMB3500S (Stockholm, Sweden) juice extractor. The resulting pulp was filtered through cheesecloth to eliminate the suspended particles and to further remove any remaining pulp.

### 2.2. Nisin, Chemicals, and Microbial Media

Nisin (Glenham Life Science Ltd., Corsham, UK) was dissolved in 0.02 mol/L of hydrochloric acid (HCl) and filtered through a 0.22 µm membrane [8]. All the chemicals and microbial media used in this study were of analytical grade.

### 2.3. Microbial Cultures and Inoculation in Tangerine Juice

*Escherichia coli* TISTR 117, *Saccharomyces cerevisiae* TISTR 5004, and *Lactiplantibacillus plantarum* TISTR 2365 were obtained from the Thailand Institute of Scientific and Technological Research (TISTR). The microbial strains were added to 10 mL of nutrient broth (NB), yeast malt broth (YMB), and MRS broth, respectively. *Escherichia coli* and *Lactiplantibacillus plantarum* tubes were incubated at 37 °C and shaken at 200 rpm for 24 h, whereas *Saccharomyces cerevisiae* tubes were incubated at 30 °C and shaken at 200 rpm for 24 h to increase the number of microorganisms (approximately 10^7^–10^8^ CFU/mL). Then, 0.1 mL was transferred into 5 mL of each broth, incubated and shaken for 24 h before being added into tangerine juice at the ratio of 1:100 (*v*/*v*). The tangerine juice had initial microbial content of approximately 10^5^–10^6^ CFU/mL. The tangerine juice was incubated at room temperature for 24 h to increase the number of microorganisms (for total plate count and yeast and mold count experiments) in the juice. The initial microbial load was approximately 10^5^–10^6^ CFU/mL.

### 2.4. UV Sterilization, Nisin Treatment, and Pasteurization of Tangerine Juice

The method of UV irradiation in tangerine juice at various doses, including 4.68, 9.36, 18.72, 37.44, 74.88, and 149.76 J/cm^2^, was previously described by Kijpatanasilp et al. [3]. The tangerine juice samples consisted of samples treated with different doses of nisin (50, 100, 150, and 200 ppm). For the pasteurization process, glass bottles containing the samples were placed in a water bath and heated to a temperature of 95 ± 1 °C for 1 min [9]. Control samples of tangerine juice without UV, nisin, and pasteurization treatment were also included.

### 2.5. UV-Irradiated Tangerine Juice for Analysis of Microbial Growth Inhibition Kinetics

The microbial analysis of the tangerine juice samples was conducted as previously described [3]. In summary, the samples were diluted with 0.1% (*w*/*v*) sterile peptone water and then subjected to microbial analysis. The results were expressed as colony-forming units per milliliter (CFU/mL) and analyzed in triplicates. The analysis utilized both zero-order and first-order kinetic models, as outlined previously by Kijpatanasilp et al. [3]. Bacterial and total microbial plates were incubated at 37 °C for 48 h, whereas yeast and mold plates were incubated at 30 °C for 48 h. Microbial calculation was conducted based on the colony counts on the media plates with the reference range of 30–300 colonies [10]. 

### 2.6. Physicochemical Properties of Nisin- and UV-Treated and Pasteurized Tangerine Juice

The pH values the total soluble solid (TSS) content were measured using a pH meter (Inobab, Tetra Con 325, Adelsdorf, Germany) and using a digital handheld refractometer (Atago No. 3840, Atago, Tokyo, Japan), respectively. Color values were assessed based on the CIE system (L*, a*, and b*), using a colorimeter (Konica Minolta, model CR-400, Tokyo, Japan). The analysis of vitamin C content involved the preparation of a standard solution of ascorbic acid (0.1%) and dichlorophenol indophenol (0.1%). The analysis of carotenoid content using solvent extraction was modified according to Jafari et al. [11,12]. Titratable acidity was analyzed following the method described by Jafari et al. in 2021 [11,12]. The Folin–Ciocalteu method was employed to determine the total phenolic compound (TPC). The TPC values were expressed in milligrams of gallic acid equivalents per liter (mg GAE/L) [11]. For the determination of the total flavonoid content (TFC), the aluminum chloride colorimetric method was used [12].

The measurement of antioxidant activity was conducted using 2,2-diphenyl-1-picrylhydrazyl (DPPH). To do so, 250 μL of the sample was mixed with 4.75 mL of DPPH methanol solution. The absorbances of both the DPPH solution and the samples were then measured at 515 nm using a spectrophotometer [13]. For the assessment of antioxidant activity using ferric reducing antioxidant power (FRAP), 4.75 mL of FRAP solution was vortex-mixed with 250 μL of the sample. The mixture was subsequently analyzed using a spectrophotometer (GENE-SYSTM 20 Visible, Thermo Fisher Scientific, Waltham, MA, USA) to measure the absorbance at 593 nm for both the FRAP solution and the samples.

### 2.7. Analysis of Microbial and Physicochemical Characterization during Storage at 4 °C

The samples consisted of various treatments, including a control sample with no treatment, UV radiation at 149.76 J/cm^2^, nisin at 200 ppm, a combined treatment with UV radiation at 149.76 J/cm^2^ and 200 ppm nisin, and a pasteurized sample treated at 95 ± 1 °C for 1 min. These samples were packaged in sealed 100 mL glass bottles and stored at a temperature of 4 °C. The physicochemical quality of the samples was assessed by measuring changes in pH, total soluble solid (TSS), titratable acidity (TA), color values, total plate count, total fungal count, and functional properties at regular intervals throughout the refrigerated storage period, as mentioned in the previous sections. The shelf life of the tangerine juice was determined by ensuring that the yeast and mold count remained below 6 log CFU/mL during storage.

### 2.8. Statistical Analysis

All experiments were carried out in triplicate and results were reported as the average ± standard deviation (SD). The data were analyzed using the analysis of variance (ANOVA) technique, utilizing the Statistical Package for Social Sciences (SPSS Version 23, USA). To assess the mean differences, Duncan’s multiple range test was applied at a significance level of *p* ≤ 0.05.

## 3. Results and Discussion

### 3.1. Impact of UV Radiation on the Microbial Growth and Inhibition Kinetics in Tangerine Juice

**Figure 1:**
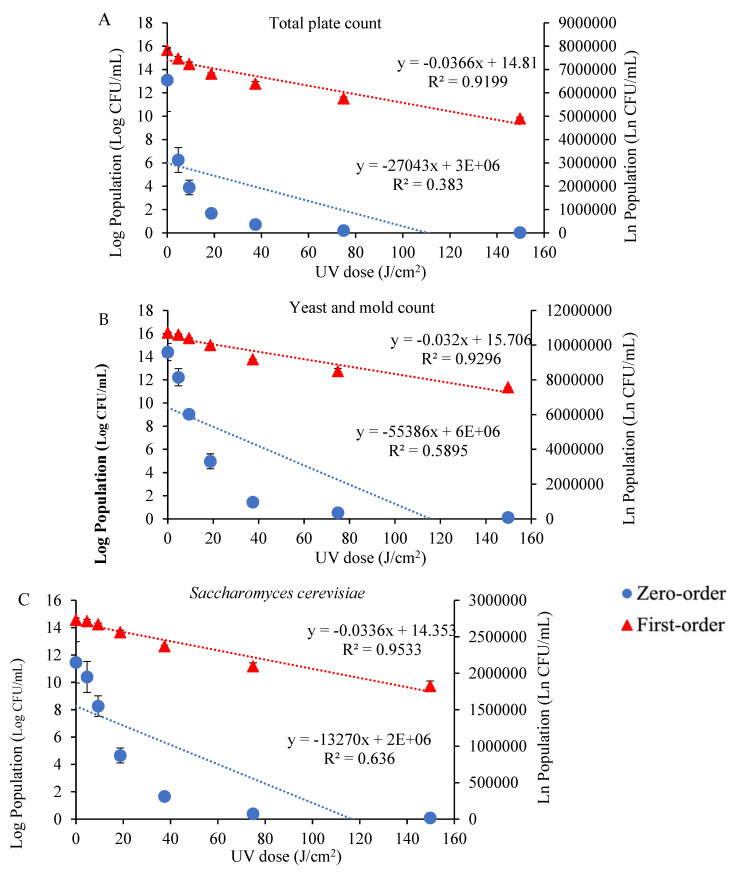

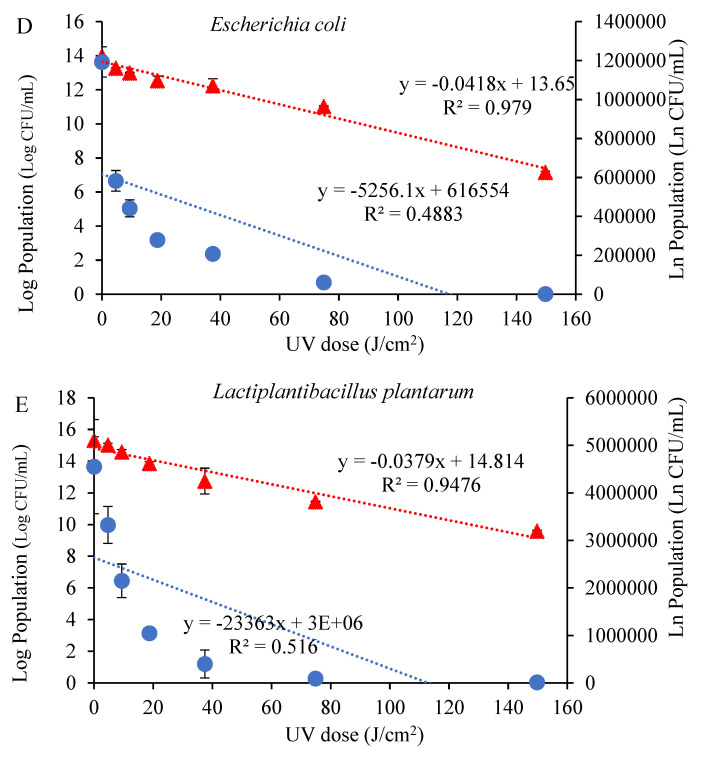
Kinetic modeling plots describing zero-order (blue circle) and first-order (red triangle) inactivation of total plate count (**A**), yeast and mold count (**B**), *Saccharomyces cerevisiae* (**C**), *Escherichia coli* (**D**), and *Lactiplantibacillus plantarum* (**E**) treated with ultraviolet radiation.

The UV treatments applied to tangerine juice for microbial inhibition are summarized in Table S1 (Supplementary Data File). Increasing the UV dose to 149.76 J/cm^2^ resulted in decreases in the total plate count and yeast and mold count to 4.26 ± 0.05 and 2.56 ± 0.04 log CFU/mL, respectively, indicating the effective reduction in microorganisms in the sample (Table S1). The populations of *E. coli*, *L. plantarum*, and *S. cerevisiae* decreased to 4.94 ± 0.02, 4.16 ± 0.03, and 4.23 ± 0.05 log CFU/mL, respectively, at the highest UV dose of 149.76 J/cm^2^. These log reduction values demonstrate the efficacy of UV treatment against the tested microorganisms. Figure 1 illustrates the inhibition kinetic plots for the total plate count (A), yeast and mold count (B), *Saccharomyces cerevisiae* (C), *Escherichia coli* (D), and *Lactiplantibacillus plantarum* (E) in tangerine juice samples exposed to various UV doses. The quantities of microorganisms in CFU/mL and in ln CFU/mL are depicted on the *y*-axis based on zero-order and first-order kinetics, respectively, while the UV dose is shown on the *x*-axis.

Table 1 presents the rate constants (k) and coefficients of determination (R^2^) for both the zero-order and first-order kinetic models applied to the total plate count, yeast and mold count, *E. coli*, *L. plantarum*, and *S. cerevisiae*. The findings indicate that the first-order model provides a relatively good fit to the experimental data, with better fitting results observed for *E. coli* compared with the other microorganisms. The rate constant values for the first-order kinetic model range from 0.0320 for yeast and mold count to 0.0418 for *E. coli*. These results suggest that the first-order model adequately describes the experimental data, with better fitting results observed for *E. coli* compared with the other microorganisms. Furthermore, the results indicate that the first-order kinetic model offers a superior fit to the experimental data when compared with the zero-order model, particularly for *E. coli*, where the first-order model exhibits a higher coefficient of determination (R^2^) and a higher rate constant (k) compared with the zero-order model.

The UV radiation induces structural changes in the genetic material, disrupting the replication and transcription processes essential for microbial growth and survival [14]. The presence of pyrimidine dimers in DNA interferes with DNA polymerase to hinder the separation of DNA strands and the accurate synthesis of new DNA strands, thereby inhibiting microbial reproduction and colony formation [15]. Initially, UV light was primarily utilized for decontaminating water and other transparent fluids. However, UV as nonthermal hurdle technology has since been shown to be effective in decontaminating a variety of liquid foods, including fruit and vegetable juices, milk, tea, coffee, liquid egg, wine, and sugar syrup [16,17].

### 3.2. Efficacy of Nisin at Different Concentrations on the Spoilage and Pathogenic Microbial Load of Tangerine Juice

The results of different concentrations (50–200 ppm) of nisin on the populations of different microorganisms, and their log reductions (log CFU/ mL), are presented in Table S2. The increase in the nisin concentration from 150 to 200 ppm reduced the microbial population of tangerine juice samples due to higher log reduction in the total plate count by 2.12 ± 0.05 log CFU/mL, yeast and mold count by 0.23 ± 0.06 log CFU/mL, *E. coli* by 1.55 ± 0.11 log CFU/mL, *L. plantarum* by 3.05 ± 0.07 log CFU/mL, and *S. cerevisiae* by 0.18 ± 0.04 log CFU/mL (*p* ≤ 0.05), compared with 50–100 ppm treatments with nisin. Generally, nisin had a greater effect on the populations of *L. plantarum* compared with the other microorganisms tested. Higher concentrations of nisin at 200 ppm showed greater log reductions in all microorganisms [18]. The mechanism of action of nisin involves several key steps that lead to microbial inhibition. In the cell wall components, nisin initially binds in the presence of certain divalent cations, such as calcium ions to the lipid II molecule, which is an essential precursor for the synthesis of peptidoglycan, a major component of the bacterial cell wall. After binding to lipid II, nisin inserts itself into the bacterial cell membrane, and it causes alterations in the lipid bilayer structure, leading to the formation of pores or ion channels to disrupt the membrane integrity of microbes [19]. The pores formed by nisin allow the uncontrolled leakage of essential intracellular components, such as ions, metabolites, and macromolecules, from the bacterial cell. This disruption of membrane integrity and loss of intracellular contents severely impairs bacterial viability. In addition, nisin also induces membrane depolarization by dissipating the electrochemical gradient across the cell membrane that potentially affects various essential cellular processes, including nutrient uptake and energy production, further compromising bacterial survival [14]. Nisin, at a concentration of 100 IU/mL in fresh apple–kale blend juice, was reported to inactivate *E. coli* K12 and *Listeria innocua* by 1.0 and 2.6 log CFU/mL, respectively [20].

### 3.3. Combined Effects of UV Irradiation and Nisin on the Spoilage and Pathogenic Microbial Load of Tangerine Juice

Table 2 displays the findings of total microbial and yeast and mold counts, as well as other microbial counts, for tangerine juice samples treated with combinations of UV radiation and nisin. The untreated control and pasteurized samples were compared with the UV, NS, and UV + NS samples. The control sample, which did not undergo any treatment, exhibited the highest total plate count (6.75 ± 0.27 log CFU/mL) among all the tested microorganisms. Furthermore, the UV and NS treatments resulted in lower microbial loads compared with the untreated control sample (*p* ≤ 0.05). However, the combined UV and nisin treatments showed the lowest total plate count (1.89 ± 0.02 log CFU/mL) and yeast and mold count (4.86 ± 0.02 log CFU/mL) compared with the UV, NS, and control samples (*p* ≤ 0.05). The pasteurization sample achieved complete log reduction with no growth of the tested microorganisms (*p* ≤ 0.05). The longan juice samples that were pasteurized and subjected to a UV dose of 149.8 J/cm^2^ exhibited greater log reduction values for total microbial, yeast/mold, and *E. coli* counts, in comparison with the untreated control and other low doses of UV radiation [21]. The combined use of UV irradiation and fumaric acid (FA) was found to be highly effective in killing *Escherichia coli* O157:H7, *Salmonella enterica* serovar Typhimurium, and *Listeria monocytogenes* in apple juice. This combined approach resulted in greater inhibition of bacterial growth in apple juice compared with using either UV or FA alone [22]. The combination of UV-C (2.52 kJ/m²) at 3 min of exposure time and nisin (15.62 μg/mL) potentially reduces *Alicyclobacillus acidoterrestris* spores and preserves ascorbic acid in orange juice [8].

### 3.4. Combined Effects of UV Irradiation and Nisin Concentrations on the Physicochemical Properties of Tangerine Juice

The effects of UV treatments (4.68–149.76 J/cm^2^) and nisin concentrations (50–200 ppm) on the physicochemical properties of tangerine juice samples are presented in Table S3. The results indicate that different doses of UV and nisin did not significantly affect the pH, TSS, TA, and color values of the tangerine juice samples (*p* > 0.05). However, significant changes were observed in the TPC, TFC, total carotenoid content, and antioxidant activity (DPPH and FRAP assays) of the tangerine juice samples treated with a UV dose of 149.76 J/cm^2^ compared with the samples treated with 50–200 ppm nisin (*p* ≤ 0.05). Subsequently, the optimized UV treatment (149.76 J/cm^2^), 200 ppm nisin (NS), and UV combined with nisin (UV + NS) were compared, as shown in Table 3. The pH values of all the treatments were similar, ranging from 3.85 to 3.87, indicating that the treatments had no significant effect on the pH of the tangerine juice (*p* > 0.05). Similarly, the TSS and TA values were similar among all treatments, suggesting that the sweetness and acidity of the juice were not negatively impacted by any of the treatments (*p* > 0.05).

Regarding color analysis, the L* values were significantly different among the treatments, with the pasteurization treatment showing the lowest L* value. The UV and NS treatments had similar L* values to the control sample (*p* > 0.05). The a* and b* values also showed significant differences among the treatments (*p* ≤ 0.05), with the UV + NS sample having the highest a* value and the pasteurization treatment having the lowest b* value. The TPC was highest in the control and UV-treated tangerine juice samples, while the pasteurized juice had the lowest TPC. Similar trends were observed for TFC, vitamin C, carotenoid content, and antioxidant activity. Overall, the UV and NS treatments, as well as the UV + NS treatment, showed insignificant effects (*p* > 0.05). In contrast, pasteurization resulted in significant reductions in TPC, TFC, vitamin C, carotenoid content, and antioxidant activity (*p* ≤ 0.05).

Antioxidant compounds have been shown to have natural protective effects against oxidative damage, commonly found in fruits, vegetables, and whole grains [23]. Pasteurization has been reported to eliminate quality-degrading microbes; however, antioxidant compounds were found to be severely affected in pineapple, mango, and watermelon juice [24]. Nisin was reported to show no notable changes to the physical and chemical properties, especially in antioxidant compounds [25]. Additionally, UV as a nonthermal treatment was reported to have less impact on the bioactive compounds that exhibit antioxidant properties in longan juice than thermal pasteurization, which led to their significant degradation [26]. Thus, optimum doses of UV (149.76 J/cm^2^), NS (200 ppm), and their combination were chosen for the storage study under refrigerated storage for 21 days.

### 3.5. Combined Effects of UV Radiation and Nisin on the Spoilage Microbial Load of Tangerine Juice during Storage at 4 °C

The control samples, which were not treated, had an initial total plate count of 3.08 ± 0.02 log CFU/mL and showed the highest increase in microbial count, reaching 9.49 ± 0.05 log CFU/mL after 21 days of storage (Table 4). Similarly, the total plate count increased over time in all samples up to 21 days, except for in the UV + NS and pasteurized samples, which showed evidence of microbial growth after day 9 and day 6 of refrigerated storage, respectively. The samples treated with UV radiation and NS alone had lower microbial counts. However, the combined treatment with UV + NS resulted in even lower microbial counts than the control, UV, and NS samples at all storage times, ranging from 0.55 ± 0.03 to 2.28 ± 0.02 log CFU/mL. Pasteurization consistently showed the lowest total microbial count throughout the storage period, ranging from 0.74 ± 0.01 to 3.11 ± 0.03 log CFU/mL.

In addition, the total microbial load obtained in the pasteurization treatment was higher compared with the UV + NS sample (21 days). The combination effect of UV and nisin treatment reported <1.7 log CFU/mL of total microbial count in tangerine juice [16]. UV treatments have been successful in deactivating spoilage microorganisms and pathogenic strains in large-scale applications for liquid foods, including juices and beverages with color and turbidity [3]. The combination of UV and nisin (UV + NS) in tangerine juice showed a synergistic effect, preserving its quality while ensuring the total plate count remained within safe consumption limits. Due to its low pH and high content of sugars and organic acids [27], the shelf life of tangerine juice was determined by its yeast and mold content not exceeding 6 log CFU/mL [28]. The yeast and mold count in the control was highest, in the range of 3.46 ± 0.04 log CFU/mL to 9.32 ± 0.13 log CFU/mL during 21 days of storage. The UV + NS combination showed a lower yeast and mold count in comparison with the untreated control tangerine juice during storage, while the NS sample exhibited a higher yeast and mold count, ranging from 3.21 ± 0.05 log CFU/mL to 9.25 ± 0.11 log CFU/mL, than that of the UV + NS treatment. Moreover, yeast and mold growth was not detected in the pasteurization treatment; however, UV + NS could be an excellent substitute to safeguard the color and bioactive properties of tangerine juice and extend shelf life for up to 9 days, while ensuring the total plate count remains within safe consumption limits. Yeast and mold are microorganisms that can grow in fruit juices and can cause spoilage by producing off-flavors, odors, and a fuzzy or cloudy appearance [20]. Refrigeration can slow down their growth; however, it has been reported that yeast and mold even showed growth in lychee juice treated with UV radiation after 35 days of storage [21]. As a result, it is important to maintain a low yeast and mold count in fruit juices to ensure their quality and safety.

### 3.6. Combined Effects of UV Radiation and Nisin on the Physicochemical Quality Changes in Tangerine Juice during Storage at 4 °C

The pH values of the control, UV, NS, UV + NS, and pasteurized tangerine juice ranged from 3.50 ± 0.01 to 3.88 ± 0.01 for all treatments during 21 days of storage at 4 °C, as shown in Table 5. The pH values slightly decreased during the entire storage period for all treatments. On day 21 of storage, the control sample exhibited the lowest pH, whereas the samples subjected to UV + NS and pasteurization treatments demonstrated the highest pH values. Total soluble solid (°Brix) values tended to decrease slightly during storage for all treatments during 21 days of storage, which was consistent with our previous study on mango and passion fruit smoothies subjected to dimethyl dicarbonate [12]. Additionally, the control sample had the lowest values of TSS, while the samples treated with UV + NS and the pasteurization sample showed the highest values on day 21 of refrigerated storage.

The titratable acidity (TA%) values slightly increased in the control, UV-, and NS-treated tangerine juice samples after 12 days of storage. The combined UV radiation and nisin treatment, along with the pasteurization treatment, showed lower TA values with insignificant differences (*p* > 0.05) compared with the other samples until the end of the storage period. The combined treatment with UV radiation and nisin showed a marked effect on maintaining the chemical quality of tangerine juice during storage at 4 °C. The L* values, which represent lightness, exhibited slight variations between the control and treated samples throughout the storage period of 0 to 21 days (Table 6). This indicates that the UV and NS treatments did not significantly affect the lightness of the tangerine juice. However, pasteurization did affect the L* values of the tangerine juice, although no significant differences were observed during the 21-day storage period. Regarding the a* values, which indicate the degree of redness or greenness, the control sample showed an initial measurement of 11.60 ± 0.12 on day 0, which increased to 12.08 ± 0.08 on day 21. The UV, UV + NS, and pasteurization treatments showed similar results, with slight increases throughout the refrigerated storage period. The NS treatment alone showed insignificant differences across all storage intervals.

In terms of the b* values, which represent the degree of yellowness or blueness, insignificant differences were observed between the control and treated samples during the entire storage period. This suggests that the treatments did not have a significant effect on the yellowness of the tangerine juice. However, a slight increase in b* values was observed in the control tangerine juice after 18 days of storage. In general, the results indicate that the combination of UV radiation and nisin treatment did not have a significant effect on the color properties of the tangerine juice samples during 21 days of cold storage. 

The total phenolic compound (TPC) of the tangerine juice decreased over time for all treatments, as shown in Table 7. Among the treatments, the most significant decrease in TPC was observed in the pasteurization treatment. The TPC decreased from 97.39 ± 1.75 mg GAE/L on day 0 to 53.54 ± 1.16 mg GAE/L on day 21. Similarly, the total flavonoid content (TFC) of the tangerine juice decreased over time for all treatments. The pasteurization treatment also showed a significant decrease in TFC, from 147.55 ± 1.01 mg QE/L on day 0 to 88.84 ± 0.74 mg QE/L on day 21. The total carotenoid content of the tangerine juice decreased over time for all treatments. The untreated control had the highest total carotenoid content of 16.6 ± 0.96 μg/100 mL on day 0, which decreased to 12.08 ± 1.28 μg/100 mL on day 21. The pasteurization sample exhibited a decrease in total carotenoid content, starting from 7.64 ± 1.06 μg/100 mL on day 0 and reaching the lowest value of 3.56 ± 0.92 μg/100 mL on day 21. In terms of vitamin C content, the combination of UV and NS treatment resulted in the most stable content during storage, with a value of 17.62 ± 1.77 mg/100 mL on day 21. In comparison, the pasteurization sample had a final value of 12.01 ± 0.75 mg/100 mL on day 21. Regarding antioxidant activity, the combination of UV + NS treatment showed a slower decrease in DPPH antioxidant activity compared with the pasteurization treatment. For the FRAP values, the UV + NS treatment exhibited higher antioxidant activity throughout the 21-day refrigerated storage period compared with the pasteurized sample.

The ascorbic acid content of the juice decreased by up to 40% during a 24-day storage period at both 4 °C and 20 °C. The storage of watermelon juice, which underwent pasteurization for 15 min, for more than 9 days had adverse effects on its color, total phenolic content, and antioxidant capacities, as reported in a study by Mandha et al. [29]. In another finding, the vitamin C content of unpasteurized mango juice decreased during storage. Furthermore, according to a study by de Oliveira Junior et al. in 2015 [23], nisin remained stable in various juices for at least 30 days at room or refrigerated temperature. 

## 4. Conclusions

The log reductions demonstrate the efficacy of UV radiation at 149.76 J/cm^2^ and pasteurization in the tested microorganisms. The first-order kinetic model inhibited microbial growth compared with the zero-order model and was more efficient. Both UV treatment and the addition of nisin significantly reduced the microbial population in tangerine juice. When UV and nisin were combined (UV + NS), no significant changes were observed in the pH, TSS, and TA values. However, notable distinctions were noted in color analysis, total phenolic compound, total flavonoid content, vitamin C and carotenoid content, as well as antioxidant activity according to DPPH and FRAP values. These results (high in functionality) have many health implications, such as helping stop or limit the damage caused by free radicals in the body and preventing them from causing damage to other cells. During storage at 4 °C, the UV + NS sample exhibited the lowest microbial load among all the samples. Furthermore, the UV + NS combination did not noticeably affect the color properties of the tangerine juice during the 21-day refrigerated storage period. While the physicochemical properties changed over time for all treatments, the antioxidant properties scored better compared with thermal pasteurization. In conclusion, the combination of UV and nisin (UV + NS) holds the potential to preserve the quality of tangerine juice during 9 days of storage at 4 °C.

## Figures and Tables

**Table 1:** Rate constant and coefficient of determination of zero-order and first-order kinetic inhibition models of microbial proliferation using UV radiation in tangerine juice.

Microorganisms	Zero-Order		First-Order	
	Rate Constant (k)	Coefficient of Determination (R^2^)	Rate Constant (k)	Coefficient of Determination (R^2^)
Total plate count	27,043	0.3830	0.0366	0.9199
Yeast and mold count	55,386	0.5895	0.0320	0.9296
*E* *. coli*	52,561	0.4883	0.0418	0.9790
*L* *. plantarum*	23,363	0.516	0.0379	0.9476
*S* *. cerevisiae*	13,270	0.6360	0.0336	0.9533

**Table 2:** Total plate count, yeast and mold count, and microbial counts of tangerine juice samples subjected to UV radiation, nisin, combined treatments with UV radiation and nisin, and pasteurization.

Sample Treatments	Total Plate Count (log CFU/mL)		Yeast and Mold Count (log CFU/mL)		*E. coli* (log CFU/mL)		*L. plantarum* (log CFU/mL)		*S. cerevisiae* (log CFU/mL)	
	Population	Log Reduction	Population	Log Reduction	Population	Log Reduction	Population	Log Reduction	Population	Log Reduction
Control	6.75 ± 0.27 ^a^	0.00 ± 0.00	6.86 ± 0.09 ^a^	0.00 ± 0.00	7.59 ± 0.02 ^a^	0.00 ± 0.00	7.85 ± 0.06 ^a^	0.00 ± 0.0	7.02 ± 0.03 ^a^	0.00 ± 0.00
UV	4.21 ± 0.05 ^b^	2.54 ± 0.04	4.84 ± 0.001 ^b^	2.02 ± 0.02	4.58 ± 0.02 ^b^	3.01 ± 0.07	5.36 ± 0.03 ^b^	2.49 ± 0.06	4.94 ± 0.16 ^c^	2.08 ± 0.05
NS	3.68 ± 0.16 ^c^	2.07 ± 0.05	6.66 ± 0.04 ^c^	0.20 ± 0.08	6.08 ± 0.30 ^c^	1.51 ± 0.09	4.81 ± 0.20 ^c^	3.04 ± 0.02	6.84 ± 0.07 ^b^	0.18 ± 0.01
UV + NS	1.89 ± 0.02 ^d^	4.86 ± 0.02	4.49 ± 0.01 ^d^	2.37 ± 0.04	2.79 ± 0.07 ^d^	4.80 ± 0.10	1.81 ± 0.01 ^b^	6.04 ± 0.15	4.73 ± 0.03 ^d^	2.29 ± 0.02
Pasteurization	0.74 ± 0.00 ^e^	6.07 ± 0.09	0.73 ± 0.01 ^e^	6.25 ± 0.02	-	6.60 ± 0.10	-	6.08 ± 0.03	-	6.02 ± 0.06

The values in the table represent the mean ± standard deviation of UV-, nisin-UV+NS, and heat-treated tangerine juice obtained from the 3 replicates. a–e indicate significant differences in each column (*p* ≤ 0.05) of the microbial experiment. Control: tangerine juice without any treatment; UV: 149.76 J/cm^2^ ultraviolet radiation dose; NS: 200 ppm of nisin; Pasteurization: tangerine juice pasteurized for 1 min at 95 ± 1 °C (come-up time was 1 min 50 s).

**Table 3:** Physiochemical characteristics of tangerine juice subjected to single and combined treatments with UV radiation, nisin, and pasteurization.

Properties	Control	UV	NS	UV + NS	Pasteurization
pH	3.87 ± 0.01 ^a^	3.87 ± 0.01 ^a^	3.86 ± 0.01 ^a^	3.85 ± 0.07 ^a^	3.87 ± 0.07 ^a^
TSS	9.0 ± 0.04 ^a^	8.9 ± 0.14 ^a^	8.9 ± 0.04 ^a^	8.9 ± 0.04 ^a^	9.0 ± 0.01 ^a^
TA	0.44 ± 0.01 ^a^	0.42 ± 0.01 ^a^	0.44 ± 0.01 ^a^	0.44 ± 0.01 ^a^	0.42 ± 0.01 ^a^
L*	71.90 ± 0.07 ^ab^	71.81 ± 0.06 ^ab^	71.94 ± 0.02 ^a^	71.76 ± 0.035 ^b^	70.95 ± 0.06 ^c^
a*	11.62 ± 0.04 ^c^	12.11 ± 0.12 ^b^	11.82 ± 0.10 ^bc^	11.91 ± 0.018 ^bc^	12.53 ± 0.10 ^a^
b*	74.73 ± 0.11 ^a^	73.70 ± 0.28 ^c^	74.26 ± 0.03 ^bc^	73.92 ± 0.046 ^b^	72.95 ± 0.12 ^d^
TPC	203.75 ± 6.38^a^	148.33 ± 2.70 ^b^	197.35 ± 4.62 ^a^	139.00 ± 1.57 ^b^	101.61 ± 13.01 ^c^
TFC	185.90 ± 0.78 ^a^	169.23 ± 3.77 ^b^	181.88 ± 4.93 ^a^	163.79 ± 3.85 ^b^	156.45 ± 2.95 ^c^
Vitamin C	41.61 ± 0.71 ^a^	36.00 ± 0.60 ^b^	41.11 ± 0.41 ^a^	35.83 ± 0.71 ^b^	19.80 ± 0.94 ^c^
Carotenoid	17.36 ± 0.38 ^a^	12.00 ± 0.30 ^b^	15.95 ± 0.11 ^a^	11.32 ± 1.94^c^	4.76 ± 0.11 ^d^
DPPH	344.09 ± 8.61 ^a^	328.36 ± 2.31 ^ab^	339.00 ± 9.26 ^ab^	322.00 ± 5.40 ^b^	292.00 ± 8.74 ^c^
FRAP	348.19 ± 3.85 ^a^	322.00 ± 3.60 ^b^	343.11 ± 1.36 ^a^	317.40 ± 3.47 ^b^	285.12 ± 6.95 ^c^

The values in the table represent the mean ± standard deviation of UV-, nisin-, and heat-treated tangerine juice obtained from the 3 replicates. a–d indicate significant differences in each row (*p* ≤ 0.05) of the physicochemical experiment. Control: tangerine juice without any treatment; UV: 149.76 J/cm^2^ ultraviolet radiation dose; NS: 200 ppm of nisin; Pasteurization: tangerine juice pasteurized for 1 min at 95 ± 1 °C (come-up time was 1 min 50 s). TSS: total soluble solid (°Brix); TA: titratable acidity (% malic acid); TPC: total phenolic compound (mg GAE/L); TFC: total flavonoid content (mg QE/L); total carotenoid content (μg/100 mL); DPPH assay (mM TE/100 mL); FRAP assay (mM TE/100 mL).

**Table 4:** Total plate count and yeast and mold count of tangerine juice subjected to optimized treatments with UV radiation, nisin and UV + NS during storage (4 °C).

Storage Time (Days)	Total Plate Count (log CFU/mL)					Yeast and Mold Count (log CFU/mL)				
	Control	UV	NS	UV + NS	Pasteurization	Control	UV	NS	UV + NS	Pasteurization
0	3.08 ± 0.02	0.29 ± 0.16	0.99 ± 0.06	-	-	3.46 ± 0.04	0.99 ± 0.06	3.21 ± 0.05	0.72 ± 0.03	-
3	4.36 ± 0.05	1.09 ± 0.13	2.21 ± 0.10	-	-	4.65 ± 0.27	1.54 ± 0.09	4.52 ± 0.14	1.20 ± 0.05	-
6	6.07 ± 0.09	1.93 ± 0.07	3.68 ± 0.08	-	0.74 ± 0.01	6.24 ± 0.64	3.68 ± 0.14	6.87 ± 0.14	3.03 ± 0.01	-
9	7.26 ± 0.05	3.83 ± 0.18	4.78 ± 0.10	0.55 ± 0.03	0.93 ± 0.03	7.85 ± 0.07	4.86 ± 0.17	7.96 ± 0.05	4.43 ± 0.02	-
12	7.60 ± 0.27	5.71 ± 0.12	6.68 ± 0.17	0.91 ± 0.01	1.54 ± 0.01	8.73 ± 0.08	6.03 ± 0.13	8.49 ± 0.02	5.71 ± 0.01	-
15	7.95 ± 0.04	6.52 ± 0.46	6.98 ± 0.04	1.44 ± 0.02	1.91 ± 0.03	9.21 ± 0.07	7.27 ± 0.041	9.04 ± 0.03	7.09 ± 0.05	-
18	8.28 ± 0.03	6.77 ± 0.23	7.66 ± 0.01	1.95 ± 0.04	2.64 ± 0.01	9.32 ± 0.13	7.96 ± 0.002	9.14 ± 0.11	7.73 ± 0.06	-
21	9.49 ± 0.05	7.24 ± 0.19	7.88 ± 0.08	2.28 ± 0.02	3.11 ± 0.03	8.99 ± 0.03	9.20 ± 0.038	9.25 ± 0.11	8.98 ± 0.02	-

The values in the table represent the mean ± standard deviation of UV-, nisin-, and heat-treated tangerine juice obtained from the 3 replicates. Control: tangerine juice without any treatment; UV: 149.76 J/cm^2^ ultraviolet radiation dose; NS: 200 ppm of nisin; Pasteurization: tangerine juice pasteurized for 1 min at 95 ± 1 °C (come-up time was 1 min 50 s).

**Table 5:** Chemical quality of tangerine juice samples subjected to combined treatments with UV radiation and nisin during storage (4°C).

Storage Time (Days)	pH					Total Soluble Solid (Brix)					Titratable Acidity (TA % Malic Acid)				
	Control	UV	NS	UV + NS	Pasteurization	Control	UV	NS	UV + NS	Pasteurization	Control	UV	NS	UV + NS	Pasteurization
0	3.88 ± 0.025	3.88 ± 0.018	3.88 ± 0.021	3.88 ± 0.001	3.88 ± 0.005	8.51 ± 0.05	9.30 ± 0.14	8.75 ± 0.01	9.30 ± 0.07	9.38 ± 0.04	0.42 ± 0.029	0.42 ± 0.029	0.42 ± 0.029	0.42 ± 0.032	0.42 ± 0.029
3	3.82 ± 0.021	3.86 ± 0.021	3.85 ± 0.025	3.86 ± 0.005	3.87 ± 0.005	8.34 ± 0.05	9.00 ± 0.01	8.60 ± 0.01	9.15 ± 0.01	9.30 ± 0.01	0.44 ± 0.011	0.43 ± 0.016	0.44 ± 0.034	0.41 ± 0.005	0.40 ± 0.001
6	3.77 ± 0.004	3.86 ± 0.032	3.83 ± 0.046	3.85 ± 0.010	3.87 ± 0.001	8.23 ± 0.04	8.70 ± 0.21	8.48 ± 0.04	8.85 ± 0.01	9.20 ± 0.01	0.49 ± 0.027	0.44 ± 0.018	0.46 ± 0.02	0.42 ± 0.005	0.40 ± 0.001
9	3.68 ± 0.007	3.85 ± 0.032	3.78 ± 0.007	3.85 ± 0.001	3.87 ± 0.005	8.05 ± 0.01	8.50 ± 0.21	8.35 ± 0.14	8.75 ± 0.01	9.05 ± 0.01	0.52 ± 0.032	0.47 ± 0.014	0.5 ± 0.029	0.44 ± 0.016	0.40 ± 0.001
12	3.59 ± 0.032	3.82 ± 0.039	3.76 ± 0.014	3.84 ± 0.005	3.87 ± 0.001	7.70 ± 0.21	8.40 ± 0.35	8.23 ± 0.25	8.65 ± 0.01	8.95 ± 0.01	0.58 ± 0.027	0.5 ± 0.007	0.53 ± 0.018	0.45 ± 0.009	0.40 ± 0.001
15	3.54 ± 0.011	3.78 ± 0.060	3.66 ± 0.011	3.81 ± 0.005	3.87 ± 0.005	7.45 ± 0.28	8.25 ± 0.35	7.95 ± 0.28	8.50 ± 0.01	8.85 ± 0.01	0.61 ± 0.036	0.52 ± 0.011	0.55 ± 0.009	0.46 ± 0.011	0.40 ± 0.001
18	3.52 ± 0.011	3.70 ± 0.021	3.62 ± 0.018	3.78 ± 0.005	3.86 ± 0.005	7.18 ± 0.18	8.03 ± 0.25	7.68 ± 0.18	8.30 ± 0.01	8.85 ± 0.01	0.63 ± 0.016	0.55 ± 0.009	0.58 ± 0.014	0.47 ± 0.009	0.4010.001
21	3.50 ± 0.011	3.66 ± 0.025	3.56 ± 0.007	3.72 ± 0.005	3.86 ± 0.001	7.11 ± 0.05	9.30 ± 0.14	8.75 ± 0.01	9.30 ± 0.07	9.38 ± 0.04	0.66 ± 0.007	0.57 ± 0.014	0.61 ± 0.018	0.49 ± 0.009	0.41 ± 0.001

The values in the table represent the mean ± standard deviation of UV-, nisin-, and heat-treated tangerine juice during refrigerated storage obtained from the 3 replicates. Control: tangerine juice without any treatment; UV: 149.76 J/cm^2^ ultraviolet radiation dose; NS: 200 ppm of nisin; Pasteurization: tangerine juice pasteurized for 1 min at 95 ± 1 °C (come-up time was 1 min 50 s).

**Table 6:** Color properties of tangerine juice samples subjected to single and combined treatments with UV radiation and nisin during storage (4°C).

Storage Time (Days)	L* Values					a* Values					b* Values				
	Control	UV	NS	UV + NS	Pasteurization	Control	UV	NS	UV + NS	Pasteurization	Control	UV	NS	UV + NS	Pasteurization
0	71.3 ± 0.04	71.0 ± 0.05	71.2 ± 0.00	71.0 ± 0.01	70.28 ± 0.09	11.6 ± 0.12	12.0 ± 0.01	11.5 ± 0.06	12.0 ± 0.01	12.5 ± 0.05	74.2 ± 0.04	73.9 ± 0.1	74.0 ± 0.02	74.0 ± 0.03	73.0 ± 0.03
3	71.4 ± 0.26	71.0 ± 0.01	71.2 ± 0.01	71.1 ± 0.03	70.12 ± 0.14	11.5 ± 0.01	12.0 ± 0.01	11.5 ± 0.01	12.1 ± 0.04	12.5 ± 0.07	74.2 ± 0.03	73.9 ± 0.2	74.1 ± 0.05	74.0 ± 0.00	73.0 ± 0.11
6	71.4 ± 0.04	71.1 ± 0.00	71.2 ± 0.18	71.1 ± 0.01	70.22 ± 0.04	11.5 ± 0.02	12.1 ± 0.01	11.6 ± 0.05	12.1 ± 0.01	12.5 ± 0.06	74.3 ± 0.00	74.1 ± 0.1	74.2 ± 0.05	74.1 ± 0.01	73.0 ± 0.01
9	71.5 ± 0.07	71.1 ± 0.05	71.4 ± 0.10	71.1 ± 0.01	70.30 ± 0.08	11.6 ± 0.15	12.1 ± 0.01	11.6 ± 0.07	12.1 ± 0.01	12.6 ± 0.09	74.5 ± 0.07	74.1 ± 0.1	74.3 ± 0.07	74.1 ± 0.01	73.1 ± 0.01
12	71.7 ± 0.11	71.2 ± 0.04	71.5 ± 0.07	71.1 ± 0.01	70.32 ± 0.07	11.9 ± 0.01	12.1 ± 0.03	11.7 ± 0.06	12.1 ± 0.03	12.6 ± 0.08	74.7 ± 0.07	74.1 ± 0.1	74.4 ± 0.11	74.1 ± 0.04	73.1 ± 0.01
15	71.7 ± 0.21	71.2 ± 0.03	71.7 ± 0.07	71.1 ± 0.01	70.33 ± 0.06	12.0 ± 0.04	12.2 ± 0.03	11.7 ± 0.03	12.1 ± 0.01	12.6 ± 0.09	74.8 ± 0.14	74.2 ± 0.1	74.6 ± 0.11	74.1 ± 0.01	73.1 ± 0.03
18	71.9 ± 0.12	71.2 ± 0.01	71.8 ± 0.04	71.1 ± 0.02	70.36 ± 0.04	12.0 ± 0.05	12.2 ± 0.02	11.8 ± 0.04	12.2 ± 0.02	12.6 ± 0.10	75.0 ± 0.07	74.3 ± 0.01	74.8 ± 0.07	74.2 ± 0.06	73.1 ± 0.04
21	72.0 ± 0.16	71.3 ± 0.04	71.9 ± 0.01	71.2 ± 0.01	70.40 ± 0.10	12.1 ± 0.08	12.2 ± 0.07	11.9 ± 0.05	12.2 ± 0.03	12.7 ± 0.07	75.4 ± 0.07	74.4 ± 0.0	75.0 ± 0.09	74.2 ± 0.01	73.2 ± 0.04

The values in the table represent the mean ± standard deviation of UV-, nisin-, and heat-treated tangerine juice during refrigerated storage obtained from the 3 replicates. Control: tangerine juice without any treatment; UV: 149.76 J/cm^2^ ultraviolet radiation dose; NS: 200 ppm of nisin; Pasteurization: tangerine juice pasteurized for 1 min at 95 ± 1 °C (come-up time was 1 min 50 s).

**Table 7:** Effects of UV radiation, nisin, UV + nisin, and pasteurization on antioxidant properties of tangerine juice during storage (4 °C).

Antioxidant Properties	Storage Time (Days)							
	0	3	6	9	12	15	18	21
**TPC** (**mg GAE**/**L**)								
Control	213.14 ± 3.40	203.04 ± 9.31	193.18 ± 8.85	189.15 ± 8.93	181.92 ± 15.01	173.29 ± 8.91	165.79 ± 14.92	160.06 ± 15.68
UV	136.53 ± 5.45	123.37 ± 11.34	114.93 ± 10.47	110.77 ± 11.34	107.45 ± 10.63	102.11 ± 6.15	95.22 ± 1.75	90.24 ± 5.34
NS	212.64 ± 2.43	196.86 ± 15.73	187.30 ± 10.80	185.31 ± 11.93	182.64 ± 10.80	176.84 ± 8.10	169.59 ± 10.80	166.38 ± 10.80
UV + NS	135.16 ± 11.61	124.74 ± 9.18	118.81 ± 5.42	109.76 ± 10.28	105.69 ± 11.88	102.98 ± 12.68	98.1 ± 13.49	86.69 ± 5.78
Pasteurization	97.39 ± 1.75	85.90 ± 4.78	76.9 ± 1.86	76.55 ± 3.7	74.70 ± 2.21	71.23 ± 1.40	60.45 ± 3.75	53.54 ± 1.16
**Total Flavonoid Content** (**mg QE**/**L**)								
Control	183.18 ± 2.68	176.67 ± 1.24	166.45 ± 0.78	165.38 ± 1.59	162.08 ± 1.52	151.07 ± 4.58	139.23 ± 0.66	129.45 ± 7.11
UV	170.19 ± 2.33	161.56 ± 0.08	156.48 ± 0.66	149.34 ± 1.2	141.53 ± 0.58	134.14 ± 1.94	123.26 ± 5.44	104.01 ± 4.78
NS	179.80 ± 0.78	171.12 ± 1.55	169.97 ± 1.17	168.76 ± 0.78	161.29 ± 1.94	142.25 ± 0.12	136.29 ± 0.39	133.40 ± 2.37
UV + NS	166.26 ± 3.15	161.48 ± 1.13	153.43 ± 2.33	147.41 ± 4.47	144.20 ± 0.47	133.15 ± 7.23	127.11 ± 7.77	99.42 ± 1.55
Pasteurization	147.55 ± 1.01	146.18 ± 0.16	144.61 ± 0.51	133.98 ± 0.62	125.71 ± 4.47	117.03 ± 1.59	106.09 ± 1.75	88.84 ± 0.74
**Total Carotenoid Content** (**μg**/**100 mL**)								
Control	16.6 ± 0.96	16.05 ± 1.01	15.18 ± 1.18	14.33 ± 2.11	13.96 ± 1.91	13.13 ± 1.57	12.8 ± 1.51	12.08 ± 1.28
UV	13.82 ± 0.98	13.13 ± 0.78	12.44 ± 1.14	11.62 ± 1.09	11.19 ± 1.14	10.31 ± 0.82	9.20 ± 0.49	8.67 ± 0.38
NS	16.22 ± 0.24	15.86 ± 0.39	14.86 ± 0.06	14.41 ± 0.28	13.9 ± 0.39	13.31 ± 0.26	12.49 ± 0.2	11.73 ± 0.10
UV + NS	14.16 ± 1.27	13.67 ± 0.84	12.66 ± 1.05	12.09 ± 0.76	11.52 ± 0.66	10.67 ± 0.09	10.04 ± 0.28	8.82 ± 0.55
Pasteurization	7.64 ± 1.06	7.04 ± 1.21	6.49 ± 1.17	6.00 ± 1.11	5.50 ± 0.98	4.65 ± 0.93	4.07 ± 1.08	3.56 ± 0.92
**Vitamin C** (**mg**/**100 mL**)								
Control	46.32 ± 2.63	42.06 ± 2.26	41.05 ± 2.26	36.48 ± 2.26	33.9 ± 0.11	32.36 ± 0.64	30.95 ± 0.38	28.77 ± 0.30
UV	35.15 ± 1.88	33.77 ± 1.28	31.32 ± 2.03	26.37 ± 1.50	24.27 ± 1.62	22.06 ± 2.26	19.96 ± 3.27	17.99 ± 1.62
NS	47.25 ± 0.94	43.02 ± 2.11	40.71 ± 1.77	36.90 ± 0.15	34.4 ± 1.05	30.31 ± 0.75	29.03 ± 0.75	26.45 ± 1.47
UV + NS	36.05 ± 1.50	34.62 ± 0.15	32.84 ± 0.04	27.97 ± 1.05	25.31 ± 1.28	21.74 ± 1.20	20.55 ± 2.29	17.62 ± 1.77
Pasteurization	28.37 ± 1.84	26.08 ± 1.02	24.56 ± 1.73	22.81 ± 0.60	18.45 ± 0.75	16.27 ± 0.75	15.31 ± 0.75	12.01 ± 0.75
**DPPH** (**mM Trolox**/**100 mL**)								
Control	392.18 ± 3.64	361.45 ± 1.82	355.18 ± 3.09	340.45 ± 7.82	323.55 ± 7.82	297.09 ± 5.45	290.82 ± 18.36	256.00 ± 1.09
UV	347.82 ± 2.31	332.91 ± 21.86	302.27 ± 1.16	278.82 ± 1.41	263.91 ± 5.01	260.45 ± 3.73	248.18 ± 1.54	226.09 ± 3.99
NS	381.27 ± 7.97	353.45 ± 3.60	347.73 ± 3.99	328.55 ± 3.60	303.64 ± 2.57	292.91 ± 3.34	283.82 ± 3.34	249.27 ± 6.43
UV + NS	333.27 ± 5.66	317.00 ± 7.07	307.45 ± 29.31	265.09 ± 7.71	254.00 ± 4.37	248.00 ± 3.60	236.82 ± 1.93	227.82 ± 5.40
Pasteurization	292.18 ± 4.37	287.09 ± 3.34	282.00 ± 5.14	233.45 ± 9.77	224.55 ± 2.57	211.82 ± 2.57	202.55 ± 4.37	170.64 ± 4.24
**FRAP** (**mM Trolox**/**100 mL**)								
Control	367.75 ± 3.51	331.09 ± 12.63	322.49 ± 0.35	304.25 ± 3.16	295.04 ± 1.23	289.68 ± 0.35	283.28 ± 0.53	272.4 ± 2.28
UV	325.65 ± 0.50	303.11 ± 2.61	296.70 ± 0.99	280.47 ± 1.12	268.19 ± 1.12	263.46 ± 2.61	255.21 ± 4.84	239.33 ± 6.2
NS	358.81 ± 2.23	331.96 ± 3.97	319.51 ± 11.91	310.74 ± 9.68	287.32 ± 3.6	280.21 ± 6.45	275.21 ± 10.3	265.21 ± 9.06
UV + NS	320.47 ± 3.10	316.61 ± 2.61	292.05 ± 2.61	273.28 ± 5.09	263.81 ± 0.37	268.19 ± 25.18	253.89 ± 11.66	226.79 ± 1.61
Pasteurization	290.56 ± 3.23	274.51 ± 6.57	268.19 ± 3.6	249.16 ± 3.47	244.33 ± 3.85	223.81 ± 5.58	220.56 ± 6.45	207.23 ± 2.48

The values in the table represent the mean ± standard deviation of UV-, nisin-, and heat-treated tangerine juice during refrigerated storage obtained from the 3 replicates. Control: tangerine juice without any treatment; UV: 149.76 J/cm^2^ ultraviolet radiation dose; NS: 200 ppm of nisin; Pasteurization: tangerine juice pasteurized for 1 min at 95 ± 1 °C (come-up time was 1 min 50 s).

## Data Availability

The data presented in this study are available on request from the corresponding author.

## Supplementary Materials

The following supporting information can be downloaded at https://www.mdpi.com/article/10.3390/foods12142725/s1. Table S1. Total plate counts, yeast and mold counts, and selected microbial counts of orange juice samples subjected to different doses of UV-radiation. Table S2. Total microbial and yeast and mold counts, and pathogenic microbial counts of orange juice samples subjected to different levels of nisin. Table S3. Physiochemical characteristics of orange juice subjected to different doses of UV-radiation and nisin.
