# Supplementary material for: Microbial Inhibition by UV Radiation Combined with Nisin and Shelf-Life Extension of Tangerine Juice during Refrigerated Storage
Source: Foods. 2023 Jul 17;12(14):2725. doi: 10.3390/foods12142725 (PMC10379832; doi:10.3390/foods12142725)
Supplement: Supplementary file 1 [file foods-12-02725-s001.zip › foods-2471699-supplementary.pdf]

Table S1. Total plate counts, yeast and mold counts, and pathogenic microbial counts of orange juice samples subjected to different doses of UV-radiation.

UV-treatments	Total plate count (log CFU/mL)		Yeast and mold count (log CFU/mL)		*E. coli* (log CFU/mL)		*L. plantarum* (log CFU/mL)		*S. cerevisiae* (log CFU/mL)	
	Population	log reduction	Population	log reduction	Population	log reduction	Population	log reduction	Population	log reduction
UV 4.68 J/cm^2	6.49±0.07 ^{a}	0.32±0.02	6.91±0.03 ^{a}	0.07±0.05	5.76±0.04 ^{a}	0.31±0.03	6.52±0.05 ^{a}	0.13±0.04	6.29±0.07 ^{a}	0.04±0.01
UV 9.36 J/cm^2	6.29±0.07 ^{ab}	0.53±0.02	6.78±0.01^{ab}	0.21±0.02	5.64±0.04 ^{ab}	0.43±0.03	6.33±0.07 ^{ab}	0.33±0.02	6.19±0.07 ^{ab}	0.14±0.02
UV 18.72 J/cm^2	5.92±0.03^b	0.89±0.13	6.52±0.06 ^{b}	0.47±0.03	5.45±0.01 ^{b}	0.64±0.25	6.02±0.04 ^{b}	0.64±0.13	5.94±0.03 ^{b}	0.39±0.00
UV 37.44 J/cm^2	5.55±0.08 ^{c}	1.26±0.01	5.99±0.01 ^{c}	1.00±0.02	5.32±0.05 ^{c}	0.77±0.22	5.53±0.36 ^{c}	1.12±0.45	5.50±0.08 ^{c}	0.85±0.03
UV 74.88 J/cm^2	5.01±0.04 ^{d}	1.81±0.05	5.54±0.10 ^{d}	1.44±0.08	4.79±0.01 ^{d}	1.29±0.11	4.97±0.015 ^{d}	1.69±0.11	4.87±0.04 ^{d}	1.47±0.16
UV 149.76 J/cm^2	4.26±0.05 ^{e}	2.56±0.04	4.94±0.00 ^{e}	2.05±0.02	3.11±0.03 ^{e}	2.97±0.46	4.16±0.022 ^{e}	2.49±0.07	4.23±0.05 ^{e}	2.10±0.21

The values in the table represent the mean ± standard deviation of UV treated orange juice obtained from the 3 replicates. $^{a\text{-}e}$ indicating significant difference in each column ($p \leq 0.05$) of the microbial experiment. UV: ultraviolet radiation doses (J/ cm^2).

Table S2. Total microbial and yeast and mold counts, and microbial counts of orange juice samples subjected to different levels of nisin.

Nisin (ppm)	Total plate count (log CFU/mL)		Yeast and mold count (log CFU/mL)		*E. coli* (log CFU/mL)		*L. plantarum* (log CFU/mL)		*S. cerevisiae* (log CFU/mL)	
	Population	log reduction	Population	log reduction	Population	log reduction	Population	log reduction	Population	log reduction
50	4.92±0.13^{a}	0.85 ±0.02	7.40±0.08 ^{a}	0.06±0.02	6.80±0.11 ^{a}	0.47±0.27	5.59±0.10 ^{a}	0.69±0.18	7.30±0.01 ^{a}	0.05±0.05
100	4.64±0.02 ^{b}	1.14±0.08	7.32±0.05 ^{a}	0.14±0.05	6.31±0.24 ^{ab}	0.96±0.14	4.14±0.09 ^{b}	2.13±0.17	7.23±0.05 ^{ab}	0.12±0.01
150	4.18±0.15 ^{c}	1.59±0.04	7.26±0.04 ^{ab}	0.19±0.06	6.02±0.20 ^{b}	1.25±0.19	3.84±0.04 ^{c}	2.43±0.04	7.21±0.05 ^{ab}	0.14±0.00
200	3.66±0.16 ^{d}	2.12±0.05	6.23±0.04 ^{b}	0.23±0.06	5.72±0.27 ^{b}	1.55±0.11	3.23±0.01 ^{d}	3.05±0.07	7.17±0.02 ^{b}	0.18±0.04

The values in the table represent the mean ± standard deviation of nisin (ppm) treated orange juice obtained from the 3 replicates. $^{a\text{-}d}$ indicating significant difference in each column ($p \leq 0.05$) of the microbial experiment.

Table S3. Physiochemical characteristics of orange juice subjected to different doses of UV-radiation and nisin.

	UV treatments						Nisin (ppm)			
Properties	4.68 J/cm^2	9.36 J/cm^2	18.72 J/cm^2	37.44 J/cm^2	74.88 J/cm^2	149.76 J/cm^2	50	100	150	200
pH	3.87±0.01 ^{a}	3.87±0.01 ^{a}	3.87±0.01 ^{a}	3.87±0.01 ^{a}	3.87±0.01 ^{a}	3.87±0.01 ^{a}	3.87±0.004 ^{a}	3.87±0.003 ^{a}	3.87±0.005 ^{a}	3.86±0.005 ^{a}
TSS	9.0±0.01 ^{a}	9.0±0.03 ^{a}	9.0±0.01 ^{a}	9.0±0.01 ^{a}	9.0±0.01 ^{a}	9.0±0.01 ^{a}	8.8±0.014 ^{a}	8.7±0.060 ^{a}	8.7±0.011 ^{a}	8.7±0.018 ^{a}
TA	0.42±0.02 ^{a}	0.42±0.01 ^{a}	0.42±0.01 ^{a}	0.42±0.01 ^{a}	0.42±0.02 ^{a}	0.42±0.02 ^{a}	0.45±0.01 ^{a}	0.45±0.01 ^{a}	0.45±0.01 ^{a}	0.45±0.01 ^{a}
L*	71.94±0.04^{a}	71.91±0.06^{a}	71.89±0.06 ^{a}	71.87±0.05^{a}	71.83±0.05^{a}	71.81±0.06^{a}	71.96±0.03^{a}	71.91 ±0.05^{a}	71.95±0.01^{a}	71.95±0.02^{a}
a*	11.86±0.06 ^{ab}	11.90±0.05 ^{a}	11.96±0.09 ^{a}	12.00±0.12 ^{a}	12.05±0.12 ^{a}	12.11±0.12 ^{a}	11.80±0.08 ^{ab}	11.81±.009^{ab}	11.82±0.09 ^{ab}	11.82±0.10 ^{ab}
b*	74.12±0.04 ^{a}	74.00±0.08 ^{a}	73.93±0.17 ^{a}	73.80±0.27 ^{ab}	73.78±0.27 ^{ab}	73.70±0.28 ^{ab}	74.26±0.02^{a}	74.26±0.05^{a}	74.27±0.02^{a}	74.27±0.04^{a}
TPC	188.08±11.85^{a}	176.00±10.04^{b}	165.48±13.74^{c}	160.06±10.50^{bc}	153.61±8.12^{c}	146.50±1.57^{d}	175.85±4.82^{b}	175.63±5.05^{b}	175.13±4.90^{b}	174.88±4.93^{b}
TFC	180.74±5.21^{b}	178.48±5.75 ^{bc}	176.51±5.67 ^{abc}	174.91±5.44 ^{bc}	172.16±3.19 ^{c}	169.23±3.77 ^{d}	202.30±4.37^{a}	199.30±5.80^{a}	198.04±5.69^{a}	197.35±4.62^{ab}
Vitamin C	39.99±2.93^{ab}	39.16±3.72^{ab}	38.55±3.31^{ab}	37.73±3.80 ^{b}	37.33±3.54 ^{b}	34.83±1.58^{c}	41.35±0.11^{a}	41.19±0.19^{a}	41.24±0.19^{a}	41.11±0.45^{a}
Carotenoids	12.71±0.49 ^{b}	13.70±0.37 ^{ab}	14.82±0.77 ^{ab}	15.68±0.14 ^{a}	15.92±0.32 ^{a}	16.21±0.12 ^{a}	16.18±0.06^{a}	16.05±0.09^{a}	16.02±0.09^{a}	15.95±0.11^{a}
DPPH	347.82±3.09^{a}	338.36±7.71^{b}	337.36±7.84^{bc}	335.73±7.07 ^{bc}	333.00±8.61^{bc}	328.36±2.31^{c}	338.45±12.21^{b}	337.82±12.1^{bc}	336.91±12.34^{bc}	336.00±12.1^{bc}
FRAP	355.04±7.07^{a}	351.88±7.07^{ab}	344.95±6.45^{b}	340.82±7.32^{bc}	328.54±6.57^{c}	317.49±3.60^{d}	346.44±1.86^{bc}	345.30±2.48^{ab}	344.42±2.73^{b}	343.11±1.36^{bc}

The values in the table represent the mean ± standard deviation of UV treated orange juice obtained from the 3 replicates. Values with different letters in the same row (a, b, c, d) are significantly different ($p \leq 0.05$). TSS: Total soluble solid (°Brix); TA: Titratable acidity (% malic acid); TPC: Total phenolic compound (mg GAE/L); TFC: Total flavonoid content (mg QE/L); Total Carotenoid (µg /100 mL); DPPH assay (mM TE/100 mL); FRAP assay (mM TE/100 mL).
